# Deep molecular modeling and mechanistic insights into type 2A von Willebrand disease with von Willebrand factor A2 domain mutations

**DOI:** 10.1016/j.rpth.2025.103233

**Published:** 2025-10-22

**Authors:** Omid Seidizadeh, Luca Mollica, Davide Giana, Luciano Baronciani, Paola Colpani, Lea Sicuro, Andrea Cairo, Flora Peyvandi

**Affiliations:** 1 Department of Pathophysiology and Transplantation, Università degli Studi di Milano, Milan, Italy; 2 Fondazione IRCCS Ca’Granda Ospedale Maggiore Policlinico, Angelo Bianchi Bonomi Hemophilia and Thrombosis Center, Milan, Italy; 3 Department of Medical Biotechnologies and Translational Medicine, Università degli Studi di Milano, Milan, Italy

**Keywords:** 2A VWD, cleavage, genetic, structural biology, VWD, type 2A, VWF

## Abstract

**Background:**

Type 2A von Willebrand disease (VWD) is characterized by impaired platelet adhesion due to the selective loss of high-molecular-weight von Willebrand factor (VWF) multimers.

**Objectives:**

To investigate A2 domain variants underlying type 2A VWD and elucidate associated disease mechanisms through comprehensive VWF assays and structural modeling.

**Methods:**

Sixty-five patients with 15 different *VWF* variants were investigated using full VWD phenotypic assays, underlying disease mechanisms, and genetic testing. Deep structural modeling (molecular dynamics, scaled molecular dynamics, and molecular docking) was employed to elucidate the effects of variants on the A2 domain.

**Results:**

Laboratory findings confirmed impaired VWF function, with reduced VWF glycoprotein Ib binding activity/VWF antigen (VWF:Ag) and VWF collagen binding/VWF:Ag ratios and increased ristocetin-induced platelet agglutination, consistent with multimer loss. Factor VIII clotting activity and VWF:Ag levels were <50 IU/dL in 45% and 58% of cases, respectively. Variants generally showed intact VWF synthesis; most patients had normal intraplatelet VWF:Ag levels; 40% had isolated reductions in intraplatelet VWF activity; 44% had reduced intraplatelet VWF:Ag and activity; and 16% were normal. VWF propeptide was normal in 80% of patients, while 89% showed elevated VWF propeptide/VWF:Ag, indicating increased clearance. Structural analyses showed that A2 variants maintained overall compactness under early shear stress, with α6 helix rigidification playing a key role in modulating interactions with the ADAMTS-13 spacer domain. All variants modestly increased solvent accessibility at the cleavage site, even in the absence of an external force.

**Conclusion:**

This integrated clinical, biochemical, and structural study reveals that A2 domain variants in type 2A VWD contribute to disease through multiple mechanisms, including impaired multimerization, altered susceptibility to proteolysis, and increased clearance.

## Essentials

• Type 2A von Willebrand disease leads to loss of large VWF multimers and bleeding.
• We studied 65 patients carrying 15 different variants in the VWF A2 domain.
• The variants contribute to disease through multiple mechanisms.
• Structural modeling revealed how A2 6-helix changes alter VWF response to shear and cleavage.

## 1 Introduction

von Willebrand factor (VWF) is a large multimeric glycoprotein (GP) that is principal for hemostasis by mediating platelet adhesion and stabilizing factor (F)VIII in circulation [1]. von Willebrand disease (VWD) is the most common inherited bleeding disorder caused by quantitative or qualitative defects in VWF. It is classified into 3 main types: type 1 (partial deficiency), type 2 (functional abnormalities, including 2A, 2B, 2M, and 2N), and type 3 (nearly complete deficiency) [2]. VWD leads to impaired platelet adhesion and/or reduced FVIII levels, and thus increased bleeding tendency, with clinical manifestations ranging from mild skin and mucocutaneous bleeding to severe episodes such as hemarthrosis and gastrointestinal hemorrhages [2,3].

Type 2A is a qualitative disorder of VWF characterized by impaired platelet adhesion due to a selective reduction or loss of high-molecular-weight multimers (HMWMs), which is the most functionally active form [2]. It has been reported that type 2A affects nearly 1.3 to 3 cases per 1000 individuals [4,5]. The pathophysiology of this subtype is primarily driven by variants in the VWF gene (*VWF*), leading to defective multimer assembly, increased VWF A2-domain susceptibility to cleavage by ADAMTS-13 (A Disintegrin and Metalloproteinase with a Thrombospondin Type 1 Motif, Member 13), or both [6,7]. Under physiological conditions, ADAMTS-13 cleaves VWF at the Tyr1605-Met1606 bond, preventing excessive platelet aggregation [2]. This cleavage can only occur if the A2 domain has been unfolded, either through tensile force or using chaotropic agents, which expose the scissile bond [[8], [9], [10]]. Several variants of the A2 domain are reported to destabilize its structure, increasing cleavage site exposure and promoting premature ADAMTS-13–mediated degradation of HMWMs even under low shear stress [[11], [12], [13]].

The spacer domain of ADAMTS-13, which adopts a single globular structure with 10 β-strands arranged in a jelly-roll topology, includes a crucial exosite that interacts with the amphipathic α6 helix of the VWF A2 domain [14]. The spacer domain plays a pivotal role in mediating this cleavage by interacting with the A2 domain [15,16]. Removal of the spacer domain from ADAMTS-13 or deletion of the α6 helix from VWF A2 reduces the cleavage rate by approximately 20-fold [17]. The A2 domain α6 helix, located in the C-terminal region of A2 (comprising the Asp1653-Arg1668 segment), is composed of 8 hydrophilic residues (6 charged and 2 uncharged) and 8 hydrophobic residues [18]. Recent discoveries highlight that the optimal extension of the α6 helix is essential for maintaining the structural integrity required for precise proteolysis [19]. Investigating the interaction between the spacer domain and the α6 helix is key to understanding type 2A VWD pathogenesis, as gene variants may disrupt this interaction, increase VWF susceptibility to cleavage, and enhance the bleeding risk. Elucidating these molecular dynamics (MD) may also uncover therapeutic targets for correcting the underlying hemostatic defect.

At our hemophilia center, more than 100 patients with the type 2A phenotype have been diagnosed, most of whom have pathogenic VWF variants in the A2 domain [20,21]. Despite this, the precise mechanisms by which A2 domain variants drive type 2A VWD remain incompletely understood. The focus of this manuscript was to investigate in depth *VWF* variants located in the A2 domain that are responsible for type 2A VWD using extensive VWD phenotypic assays and structural biology. We further aimed to investigate the underlying disease mechanisms in this type 2A phenotype.

## 2 Methods

### 2.1 Patients

We included all genetically confirmed type 2A patients with variants located in the VWF A2 domain (ie, *VWF* exon 28) and referred to the A. Bianchi Bonomi Hemophilia and Thrombosis Center in Milan. Informed consent was obtained from all patients according to the Declaration of Helsinki, and the study was approved by the Board of the Fondazione IRCCS Ca’ Granda Ospedale Maggiore Policlinico, Milan. All patients were extensively investigated regarding clinical manifestations, VWD biochemical phenotypic tests, and genetic studies in order to achieve a correct diagnosis [20].

### 2.2 Laboratory phenotype evaluation

Laboratory assays for VWD diagnosis have been previously described [20]. Briefly, VWF antigen (VWF:Ag) levels were measured by immunoturbidometric assay, and platelet-dependent VWF GPIb binding activity (VWF:GPIbR) was assessed using the ristocetin cofactor activity assay (HemosIL, Instrumentation Laboratory). FVIII clotting activity (FVIII:C) was determined by a one-stage assay, while VWF collagen binding (VWF:CB) was measured with collagen type I or III (or a combination) using an enzyme-linked immunosorbent assay method. The ristocetin-induced platelet agglutination (RIPA) assay was performed as previously described [22]. Intraplatelet VWF:Ag and VWF activity levels were measured as described [23,24]. Briefly, platelet-rich plasma was centrifuged at 1000 × *g* for 10 minutes to obtain a platelet pellet, and resuspended in Tris-HCl buffer with Protease Inhibitor Cocktail I (Calbiochem, Merck). Platelets were then lysed with 0.5% Triton X-100 (Rohm and Haas) and subjected to repeated snap-freeze-thaw cycles in liquid nitrogen. To further investigate the underlying disease mechanisms in type 2A patients, in addition to platelet VWF assays, we measured VWF propeptide (VWFpp) levels and the ratios of FVIII:C/VWF:Ag and VWFpp/VWF:Ag. The VWFpp antigen and FVIII:C/VWF:Ag ratio were used to evaluate decreased VWF synthesis/secretion [21], and the VWFpp/VWF:Ag ratio was used to evaluate enhanced VWF clearance, as previously described [25,26].

### 2.3 Genotype analysis

Genetic analysis was performed using either polymerase chain reaction/Sanger sequencing or next-generation sequencing. For Sanger sequencing, *VWF* was sequenced to identify candidate variants by amplifying exons encoding specific VWF domains, including intron-exon boundaries [20]. For next-generation sequencing, the entire *VWF* gene (coding regions, exon-intron boundaries, and the 5' and 3' untranslated regions) was sequenced. We also collected data on ClinVar (Clinical Variation public archive database) classification, gnomAD (The Genome Aggregation Database) variant frequency, and CADD (Combined Annotation-Dependent Depletion) scores for the identified variants. ClinVar provides clinical significance based on expert classifications; gnomAD helps assess population frequency to distinguish rare disease-causing variants from benign polymorphisms; and CADD predicts the functional impact of variants. Combining these metrics improves accuracy in identifying pathogenic variants [27].

### 2.4 MD and scaled MD

**Figure 1:**
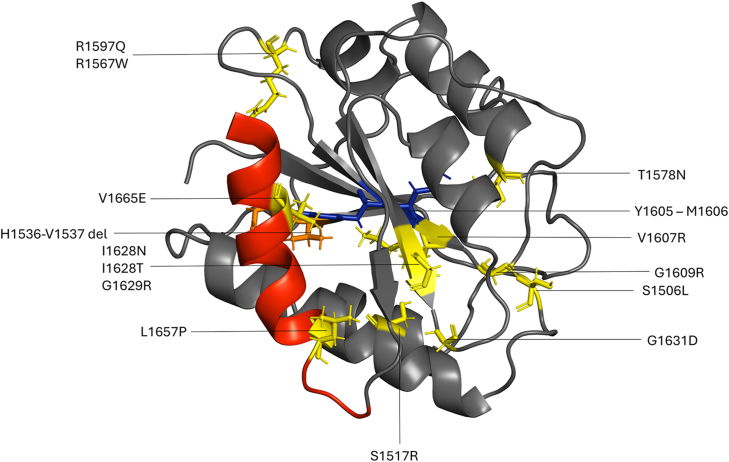
Ribbon representation of the structure of the von Willebrand factor A2 domain. The mutated residues are reported as sticks and explicitly indicated by their sequence number. The α6 helix is colored red, and the location of the ADAMTS-13 cleavage site is colored blue. ADAMTS-13, A Disintegrin and Metalloproteinase with a Thrombospondin Type 1 Motif, Member 13.

The structure of the wild-type (WT) A2 domain, spanning residues 1495 to 1671, was obtained from the Protein Data Bank (PDB ID: 3GXB) [18]. All mutants (Figure 1) were generated and energetically minimized using the FoldX suite [28]. In total, 15 structures were used to perform plain MD and scaled MD (SMD) [29] simulations using the GROMACS suite of programs (version 2020.1) [30] and its implementation in the BiKi software package [31], respectively. All systems were protonated and parametrized using the AMBER99SB-ILDN force field standards [32].

A parallelepipedal solvent box made of 10,000 water molecules (TIP3P33) [33] was created around each protein. The overall system charge was balanced with several counterions (Na^+^), depending on the net charge of the protein and the type of mutation (the charge of WT A2 is −10). After minimization using the steepest descent method (with a convergence of the total force equal to 100 kJ mol^–1^ nm^–1^), the system was equilibrated (with isotropic positional restraints on protein heavy atoms, *k* = 1000 kJ mol^–1^ nm^–2^) for 500 ps in the *NPT* ensemble (*p* = 1 atm and *T* = 300 K), then for 500 ps in the *NVT* ensemble (*T* = 300 K) [34]. We then performed a 100 ns MD or SMD simulation for each system in the *NVT* ensemble employing a timestep of 2 fs and constraining all covalent bond lengths with LINCS, with a scaling factor of λ = 0.8 for SMD [35].

### 2.5 Molecular docking

The docking calculations between A2 and spacer structures were performed using the HADDOCK software [36], a collection of Python and Crystallography and NMR System [37] scripts. These allowed data-driven rigid-body docking calculations, ie, no internal motions of the single domains were allowed while docked, followed by flexible refinement. Twenty-one ambiguous combinatorial restraints were used (minimum, 3.5 Å; maximum, 5.5 Å) between the sidechains’ functional groups of residues reported in the literature (A2: R660, Y661, and Y665; spacer: E1660-R1668) [38] during the rigid-body docking step. The flexibility of the complex interface was then allowed to relax the resulting complexes, yielding 100 final structures for each A2 spacer system using an implicit solvent model and parametrizing the interactions with the OPLS force field. The procedure was repeated with structures obtained from MD and SMD simulations, ie, we also performed MD and SMD simulations for the spacer domain, as reported above. An estimate of the energetics of the complexes, ie, their binding affinities (free energies), was obtained using the molecular mechanics Poisson–Boltzmann surface area (MM-PBSA) method [39]. This was implemented in the gmx_mmpbsa tool [40], and applied to all the complex structures obtained, as previously described, and assembled into an MD-like trajectory. This tool applies the charge hydration asymmetry model to compute atomic radii [41], and a ratio of 4 between the longest dimension of the rectangular finite-difference grid and the solute was adopted.

### 2.6 Statistical analysis

Continuous variables were described as median (range), and categorical variables as counts (percentages). Statistical analyses were performed with the R statistical software environment (R Foundation for Statistical Computing). The Mann–Whitney U-test was used to compare medians between 2 independent groups, and a *P* value < .05 was considered statistically significant.

## 3 Results

### 3.1 Type 2A patients

In a cohort of 371 patients with type 2 VWD, 105 (26%) were confirmed type 2A based on genetic testing. Among these, 65 patients (62%) carry a VWF variant located in the A2 domain, associated with the type 2A (IIA) phenotype. This specific variant leads to an increased susceptibility of the A2 domain to cleavage by ADAMTS-13 and/or defective multimerization [3].

### 3.2 Laboratory phenotype results

**Figure 2:**
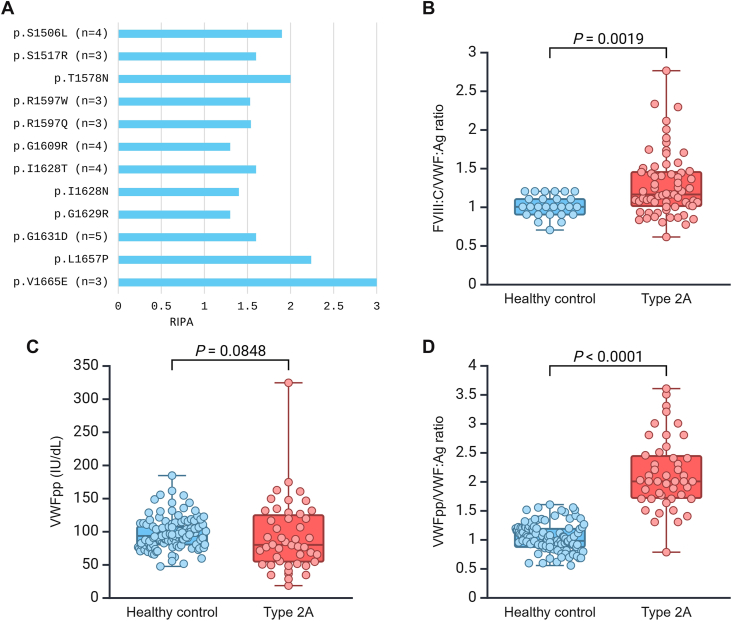
Ristocetin-induced platelet agglutination results and underlying disease mechanisms. (A) Ristocetin-induced platelet agglutination was performed in 32 patients, representing 12 of the 15 identified *VWF* variants. (B, C) The factor VIII activity (FVIII:C)/von Willebrand factor (VWF) antigen (VWF:Ag) ratio and VWF propeptide (VWFpp) levels were assessed to evaluate impaired VWF synthesis and/or secretion. (D) The VWFpp/VWF:Ag ratio was used to investigate increased VWF clearance. Created with BioRender.com. IU, International Units.

Table 1 presents laboratory assay results for the cohort (*N* = 65). These findings align with the typical features of type 2A: a loss of HMWM (Supplementary Figure) plus severely reduced VWF:GPIbR/VWF:Ag and VWF:CB/VWF:Ag ratios (median, 0.25 and 0.14, respectively). The cohort exhibited a broad range of FVIII:C levels (21-100 IU/dL), with a median of 51 IU/dL, and 45% of cases had levels < 50 IU/dL. Similarly, VWF:Ag levels varied widely, ranging from 7 to 130 IU/dL, with a median of 41 IU/dL, and 58% had levels < 50 IU/dL. RIPA results were available for 32 patients (corresponding to 12 of the 15 different *VWF* variants identified). All patients exhibited elevated ristocetin requirements for platelet agglutination in the RIPA assay, consistently exceeding the normal range of 0.8 to 1.2 mg/mL (Figure 2A).

**Table 1:** Laboratory tests in patients with type 2A von Willebrand disease.

Laboratory tests	Type 2A VWD, median (range)
FVIII:C IU/dL	51 (21-100)
VWF:Ag IU/dL	41 (7-130)
VWF:GPIbR IU/dL	10 (3-29)
VWF:GPIbR/VWF:Ag	0.25 (0.04-0.65)
VWF:CB IU/dL	6 (1-26)
VWF:CB/VWF:Ag	0.14 (0.02-0.42)
RIPA (mg/mL)^a^	1.6 (1.3-3)
VWFpp IU/dL^b^	80 (18-324)
VWFpp/VWF:Ag^b^	2 (0.78-3.6)

Values are presented as median (range).

FVIII:C, factor VIII activity; RIPA, ristocetin-induced platelet agglutination; VWD, von Willebrand disease; VWF:Ag, von Willebrand factor antigen; VWF:CB, von Willebrand factor collagen binding; VWF:GPIbR, von Willebrand factor glycoprotein Ib binding assay using recombinant glycoprotein Ib; VWFpp, von Willebrand factor propeptide.

a RIPA was available for 32 cases.

b VWFpp and the VWFpp/VWF:Ag ratio were available for 46 cases.

### 3.3 Molecular genetic results

We identified 15 different *VWF* variants associated with type 2A, all located within the A2 domain (Table 2). All variants were missense except for 1 in-frame deletion (p.His1536_Val1537del). The CADD scores were consistently >20 (except for 1 variant), suggesting the pathogenic nature of these variants. The ClinVar classifications were either pathogenic or likely pathogenic, except for 1 variant, which was classified as of uncertain significance. Among these 15 variants, 11 were absent in the gnomAD population (807,162 total individuals), and the remaining 4, which were present in gnomAD, had a frequency < 0.0001, indicating the extreme rarity of all 15 variants.

**Table 2:** Identified variants in the A2 domain of von Willebrand factor associated with type 2A von Willebrand disease.

Exon	cDNA	Protein (no. of patients)	ClinVar	CADD score	MAF in gnomAD
28	c.4517C>T	p.Ser1506Leu (*n* = 7)	Pathogenic	32	Not found
28	c.4551C>G	p.Ser1517Arg (*n* = 3)	Likely pathogenic	22.4	Not found
28	c.4606_4611delCACGTC	p.His1536_Val1537del (*n* = 1)	Pathogenic	-	Not found
28	c.4790G>A	p.Arg1597Gln (*n* = 9)	Pathogenic	24.1	Not found
28	c.4789C>T	p.Arg1597Trp (*n* = 3)	Pathogenic	28.3	6.196e-7
28	c.4820T>A	p.Val1607Asp (*n* = 1)	Likely pathogenic	29.0	Not found
28	c.4825G>A	p.Gly1609Arg (*n* = 1)	Pathogenic	23.7	0.000003098
28	c.4883T>C	p.Ile1628Thr (*n* = 10)	Pathogenic	23.8	0.000001859
28	c.4883T>A	p.Ile1628Asn (*n* = 1)	Pathogenic	27.7	Not found
28	c.4885G>C	p.Gly1629Arg (*n* = 11)	Likely pathogenic	24.3	Not found
28	c.4892G>A	p.Gly1631Asp (*n* = 10)	Likely pathogenic	25.4	Not found
28	c.4970T>C	p.Leu1657Pro (*n* = 2)	Likely pathogenic	24.2	Not found
28	c.4994T>A	p.Val1665Glu (*n* = 3)	Likely pathogenic	23.9	Not found
28	c.5014G>A	p.Gly1672Arg (*n* = 1)	Uncertain significance	9.446	0.0001879
28	c.4733C>A	p.Thr1578Asn (*n* = 1)	Pathogenic	27.1	Not found

CADD, Combined Annotation-Dependent Depletion; cDNA, complementary DNA; ClinVar, Clinical Variation public archive database; gnomAD, Genome Aggregation Database; MAF, Minor Allele Frequency.

### 3.4 Underlying mechanisms of type 2A VWD

#### 3.4.1 Intraplatelet VWF

**Figure 3:**
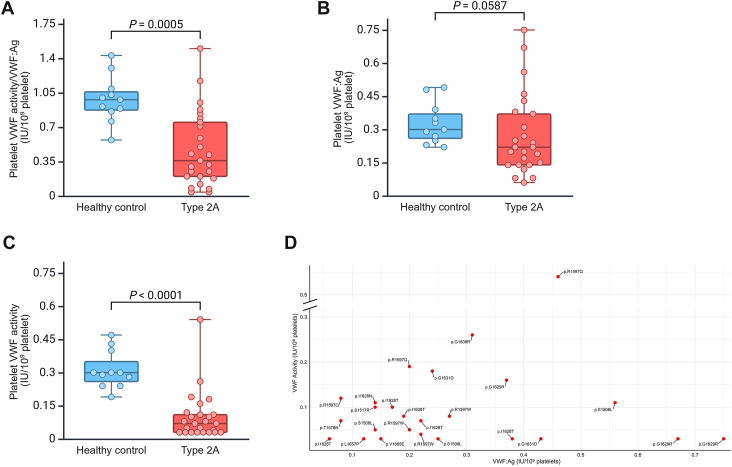
Intraplatelet von Willebrand factor (VWF) measurements. (A) Ratio of intraplatelet VWF activity to intraplatelet VWF antigen (VWF:Ag). (B) Intraplatelet VWF:Ag levels. (C) Intraplatelet VWF activity levels. (D) Correlation between intraplatelet VWF:Ag and VWF activity for each identified *VWF* variant. Created with BioRender.com. IU, International Units.

In a subset of 25 patients, comprising 12 out of the 15 identified *VWF* variants, we measured intraplatelet VWF:Ag and VWF activity. An abnormal VWF activity/VWF:Ag ratio (<0.7) was observed in 17 cases, and a normal ratio with proportionally reduced levels was found in 4 cases (Figure 3A). Compared with our normal ranges (0.22-0.6 IU/10^9^ platelets for VWF:Ag and 0.15-0.58 IU/10^9^ platelets for VWF activity), 10 patients (40%) exhibited only abnormal VWF activity, while in 11 cases (44%), both VWF:Ag and VWF activity were reduced. In the remaining 4 patients (16%), we found both VWF:Ag and VWF activity at normal levels. There was no significant difference in the median VWF:Ag between patients (*n* = 25; 0.22) and healthy controls (*n* = 11; 0.3; *P* = .0587; Figure 3B). However, there was a significant difference in the median VWF activity between patients and controls (0.07 vs 0.3; *P* < .0001; Figure 3C). Overall, there was a significant difference between patients and controls in the VWF activity/VWF:Ag ratio (0.4 vs 0.98; *P* = .0005; Figure 3A). The results of intraplatelet VWF:Ag and VWF activity for each variant are shown in Figure 3D.

#### 3.4.2 VWF synthesis/secretion and clearance

We used the FVIII:C/VWF:Ag ratio and VWFpp levels as markers of VWF synthesis/secretion, and the VWFpp/VWF:Ag ratio as a marker of enhanced clearance. Type 2A (*n* = 65) cases had a significantly higher FVIII:C/VWF:Ag ratio than healthy controls (*n* = 26; median, 1.2 vs 1.0; *P* = .0019; Figure 2B). In contrast, median VWFpp (*n* = 46) levels did not differ significantly between patients and controls (80 vs 93 IU/dL; *P* = .0848), with only 20% showing values <50 IU/dL (Figure 2C). The VWFpp/VWF:Ag ratio was significantly higher in patients (median, 2.0 vs 0.98; *P* < .0001), indicating increased VWF clearance (Figure 2D). Among these 46 cases with available VWFpp/VWF:Ag data, 89% showed evidence of enhanced clearance (ratio > 1.6).

### 3.5 MD simulations

Plain MD simulations allow sampling of the structural and dynamical properties of macromolecules at a given temperature by following their evolution on the physical potential energy surface, thereby enabling the detection of structural perturbations introduced by sequence mutations. In contrast, SMD modifies the potential energy surface, softens interactions, and facilitates conformational transitions, such as unfolding or unbinding, that would otherwise require simulation times beyond practical computational limits. In the present study, SMD was used as a proxy to capture the early perturbation stages prior to proteolysis of the VWF A2 domain under external forces. As viscous stresses energetically favor the population of unfolded states [42], SMD allows the observation of nonequilibrium protein states under shear stress conditions in circulating blood following vascular injury.

**Figure 4:**
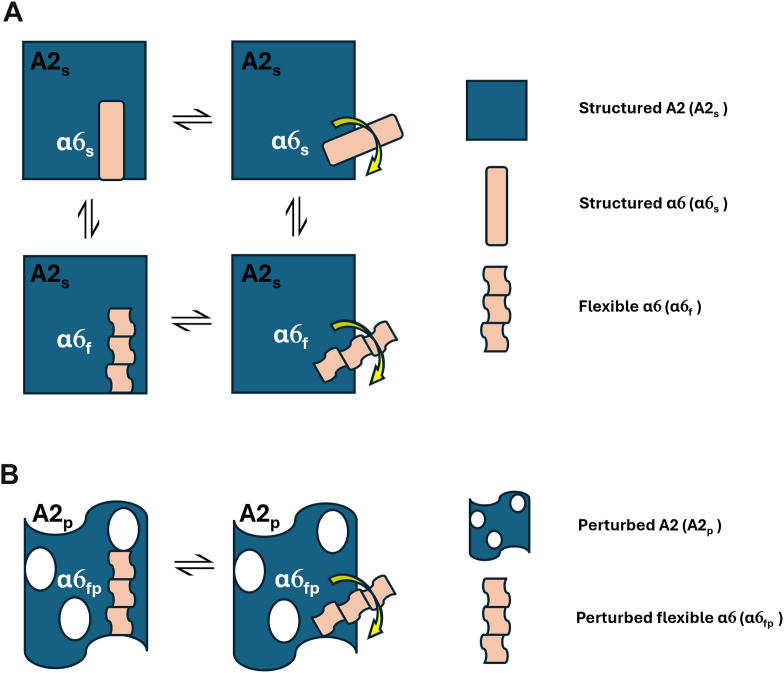
Scheme of interpretative models adopted in the present study for the inspection of the outcomes of (A) molecular dynamics and (B) scaled molecular dynamics simulations.

To assess the behavior of the mutated A2 domain, focusing on the role of the α6 helix, we proposed 2 simplified models. The unperturbed model (Figure 4A) allows the α6 helix to retain its structure or gain flexibility, with or without maintaining native contact. The perturbed model (Figure 4B) assumes that the α6 helix becomes flexible and fluctuates between compact and protruding conformations. To explore these dynamics, we analyzed 4 parameters that reflect the overall protein stability and specific behavior of the α6 helix:

- α6 Internal contacts, ie, the overall number of atom–atom contacts between each residue belonging to the α6 helix.
- α6 Contacts with all A2 parts differently from itself, ie, the overall number of atom–atom contacts between each residue belonging to the α6 helix and the residues belonging to all the A2 structures, apart from the α6 helix.
- A2 backbone atoms root mean square deviation (RMSD), ie, the measurement of the similarity between the MD/SMD frames and the starting structure (as deposited in the PDB or modeled based on it).
- A2 solvent accessible surface area (SASA), ie, the amount of protein surface exposed to the solvent.

**Figure 5:**
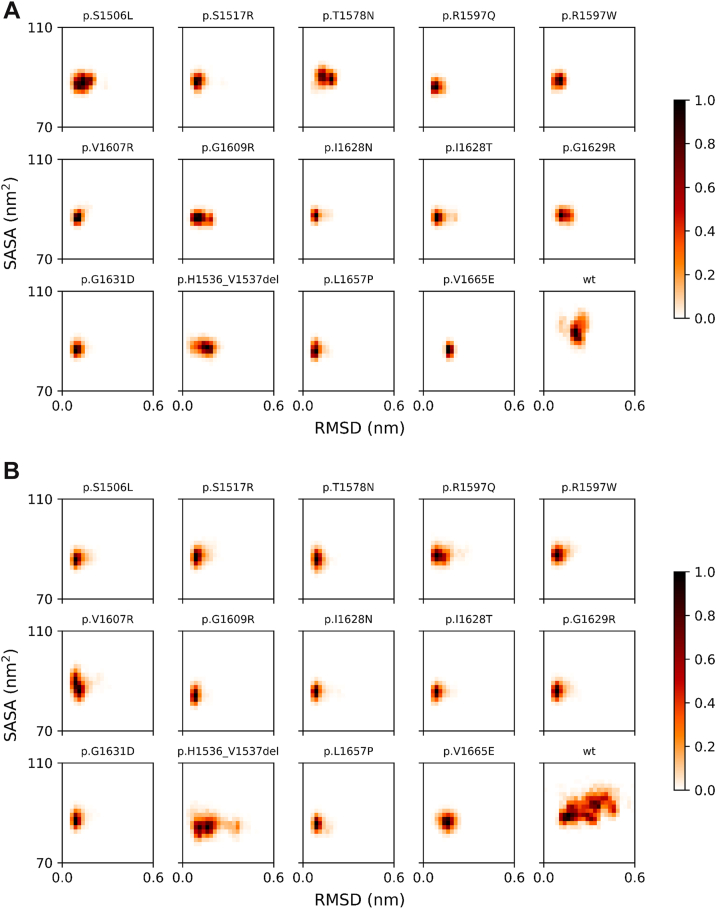
Correlation maps of A2 backbone atom root mean square deviation (RMSD) with A2 solvent accessible surface area (SASA). (A) Plain molecular dynamics and (B) scaled molecular dynamics (λ = 0.8). Populations are normalized to their maximum value of 1. WT, wild-type.

The correlation between these measurements effectively revealed structural and dynamic changes in the protein caused by variants. Maps showing the relationship between backbone RMSDs and SASAs provided an efficient overview of A2 domain dynamics in response to molecular perturbations. Plain MD simulations (Figure 5A) showed that, despite the high RMSD similarity, which indicates overall protein stability across all species, the amount of the protein’s accessible surface was higher in WT A2 than in mutants, suggesting a naturally more dynamic behavior of the former, even in nonperturbed conditions. Once perturbation was introduced by means of SMD (Figure 5B), only the WT domain was strongly influenced, leaving the average SASA substantially unaltered but largely spreading the RMSD toward higher values, suggesting a more flexible and disordered state not present in any of the investigated mutants.

**Figure 6:**
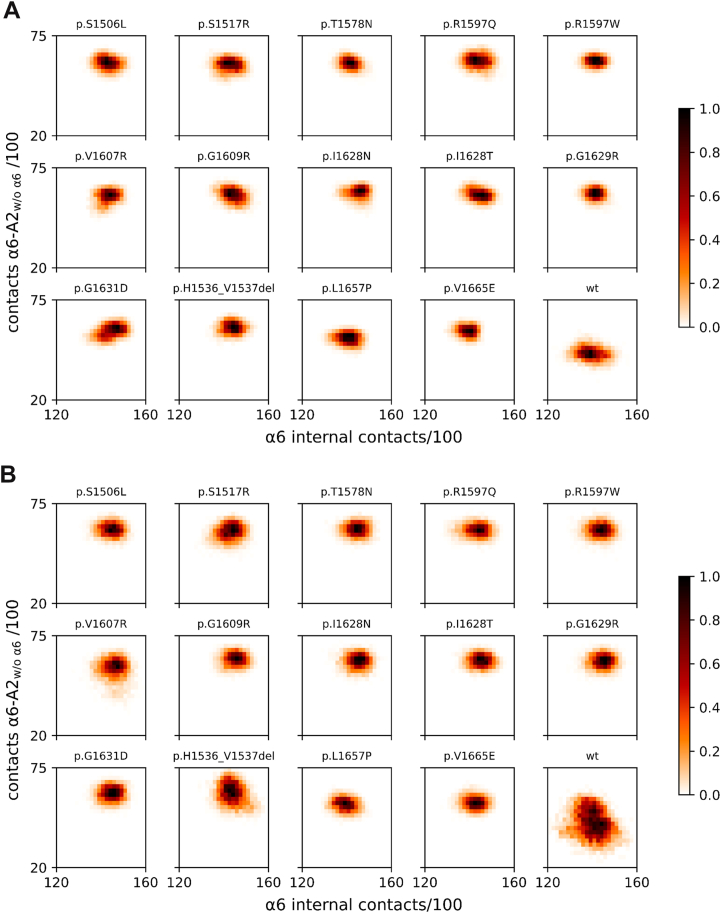
Correlation maps of α6 internal contacts with α6-A2 (ie, A2 = A2 without α6) contacts. (A) Plain molecular dynamics and (B) scaled molecular dynamics (λ = 0.8). Populations are normalized to their maximum value of 1. A2_w/o a6_, A2 without alpha6 helix; WT, wild-type.

The relationship between the α6 internal contacts and the rest of the A2 domain highlights again a unique behavior of the WT domain in plain MD simulations (Figure 6A). The average number of internal contacts of the α6 helix remained unchanged across all species (despite a slightly larger spread in the WT form), whereas the α6-A2_w/o α6_ contacts were lower in the WT form than in the rest of the inspected molecules. The same pattern was observed once a perturbation was introduced (Figure 6B), leading to local α6 disorder and loss of structure.

**Figure 7:**
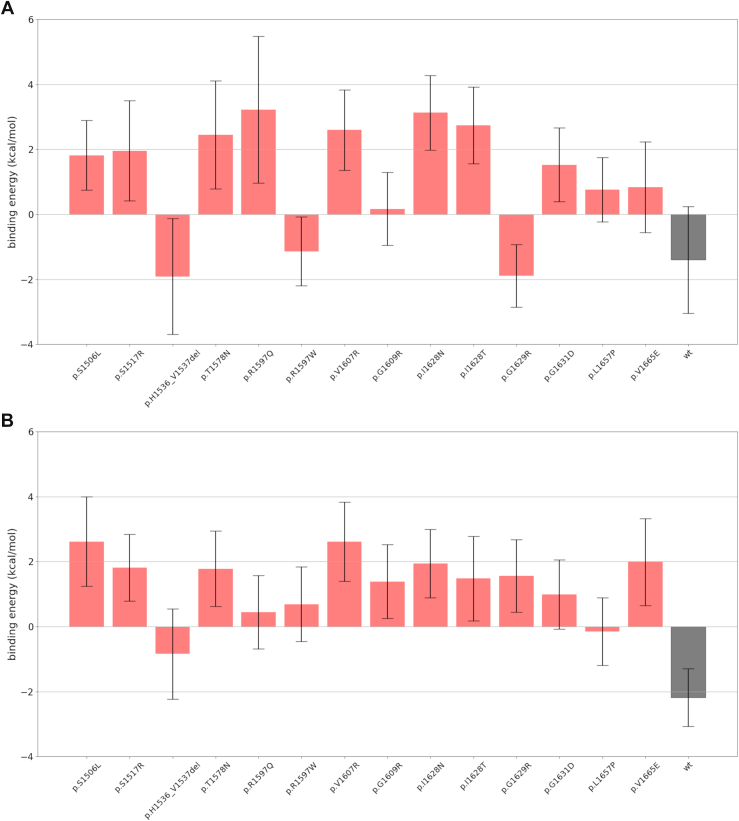
Docking-based molecular mechanics Poisson–Boltzmann surface area binding energy of the A2-spacer complex formed by (A) molecular dynamics-sampled structures and (B) scaled molecular dynamics-sampled structures. The energy is reported in kcal/mol, and the error bars represent the SE of the estimate. WT, wild-type.

Molecular docking of A2 and the spacer domain, under both unperturbed (MD) and perturbed (SMD) conditions, followed by MM-PBSA calculations, revealed consistently weak or unfavorable interactions (Figure 7). In MD-based complexes, WT A2 and most mutants showed little to no affinity, while only p.His1536_Val1537del, p.Arg1597Trp, and p.Gly1629Arg retained weak binding. Upon SMD-induced unfolding, favorable binding was observed exclusively in WT A2 (−2.1 kcal/mol), whereas all mutants exhibited absent or unfavorable interactions. In parallel, mutant systems displayed a modest increase in SASA at the proteolytic cleavage site, even under plain MD conditions (Table 3).

**Table 3:** Solvent accessible surface area (SASA_cs_) of the A2 cleavage site (residues 1603-1606) for the metalloprotease domain of ADAMTS-13 (A Disintegrin and Metalloproteinase with a Thrombospondin Type 1 Motif, Member 13) in molecular dynamics and scaled molecular dynamics simulations. Corresponding SEs and the variation in SASA_cs_ with respect to the wild-type structure (ΔSASA_cs.wt_) are reported as well.

Variants	MD			SMD		
	SASA_cs_, nm^2^	SE	DSASA_cs,wt_, %	SASA_cs_, nm^2^	SE	DSASA_cs,wt_, %
WT	7.60	0.02	-	7.63	0.03	-
p.H1536_V1537del	8.05	0.03	6	7.93	0.02	4
p.G1629R	7.70	0.03	1	7.71	0.03	1
p.G1631D	7.64	0.03	1	7.78	0.02	2
p.G1609R	7.67	0.03	1	7.74	0.02	2
p.I1628N	7.75	0.03	2	7.69	0.03	1
p.I1628T	7.72	0.02	2	7.62	0.03	0
p.L1657P	7.71	0.02	2	7.67	0.03	1
p.R1597Q	7.76	0.02	2	7.81	0.03	2
p.R1597W	7.73	0.03	2	7.70	0.03	1
p.S1506L	7.80	0.03	3	7.70	0.02	1
p.S1517R	7.72	0.03	2	7.66	0.03	0
p.T1578N	7.74	0.02	2	7.71	0.03	1
p.V1665E	7.75	0.03	2	7.76	0.03	2
p.V1607R	7.69	0.02	1	7.68	0.03	1

MD, molecular dynamics; SMD, scaled molecular dynamics; WT, wild-type. SASA_cs_, Solvent accessible surface area of the A2 cleavage site. ΔSASAcs.wt represents the change in SASAcs relative to the wild-type structure.

## 4 Discussion

In this comprehensive study, we present a detailed phenotypic and structural characterization of patients with type 2A VWD carrying variants in the VWF A2 domain. Our cohort comprised 65 genetically confirmed cases, representing one of the largest collections of this subtype. The A2 domain variants identified in this study clustered around regions known to affect the multimerization process and susceptibility to proteolysis, aligning with the well-established mechanisms underlying the historically described “2A (IIA)” phenotype. This particular phenotype represents the most common form of type 2A, as reported in studies from Spain [43], France [44], and Italy [20].

Laboratory assessments confirmed the expected pattern of impaired VWF functions, reflected by disproportionately reduced VWF:GPIbR/VWF:Ag and VWF:CB/VWF:Ag ratios due to loss of VWF HMWMs. Our study demonstrated wide variability in FVIII:C and VWF:Ag levels in type 2A, with 45% and 58% of patients, respectively, showing levels below 50 IU/dL, perhaps contributing to further bleeding risk. Indeed, type 2A is generally associated with the most severe phenotype among the type 2 subtypes [7,11,45], which may be partially explained by reduced FVIII:C levels in addition to the loss of HMWMs. Notably, all RIPA showed reduced platelet agglutination, requiring supranormal ristocetin, consistent with loss of functional VWF multimers.

Intraplatelet VWF assays (VWF:Ag and activity) are useful for understanding the pathophysiology of VWD, distinguishing between decreased VWF synthesis and increased VWF clearance, thus aiding in the diagnosis and subtyping of VWD [23,46]. Our intraplatelet VWF analysis of a subset of 25 type 2A patients showed significant impairment at the platelet level. Although the total intraplatelet VWF:Ag levels did not significantly differ from controls, VWF activity and the VWF activity/VWF:Ag ratio were both markedly reduced in most patients, with 68% showing ratios < 0.7 and 44% displaying parallel reductions in both VWF:Ag and VWF activity. These findings highlight that platelet VWF can be quantitatively preserved but functionally defective in most type 2A cases, consistent with the known pathophysiology involving defective multimerization and enhanced susceptibility to ADAMTS-13–mediated cleavage. Conversely, in 16% of cases, both intraplatelet VWF:Ag and VWF activity were within normal ranges, suggesting that in these cases, the primary disease mechanism involves increased proteolytic cleavage by ADAMTS-13 rather than defective multimerization. Historically, the type 2A phenotype was subtyped into group 1, which involves both impaired VWF multimer assembly and increased proteolysis, and group 2, resulting from increased proteolysis. Thus, both show reduced VWF function, but platelet VWF:Ag is reduced or normal in group 1 and typically normal in group 2 [6,[47], [48], [49]]. In our study, while some variants aligned with the traditional group 1 or group 2 classification, others did not clearly fit into either category. We also found variable platelet VWF results for the same type 2A variant (Figure 3D). This highlights type 2A heterogeneity and the value of intraplatelet VWF assays beyond plasma measurements.

We found no significant difference in patients’ VWFpp compared with controls, suggesting normal synthesis in 80% of patients and confirming intraplatelet VWF assays. However, the FVIII:C/VWF:Ag ratio did not show such results and was overall elevated. Conversely, an elevated VWFpp/VWF:Ag ratio in our cohort confirmed increased VWF clearance, consistent with our earlier findings [25] and previous reports [50], thus indicating that enhanced clearance is an additional common feature of many type 2A variants. Collectively, our results from platelet and plasma VWF support a model in which type 2A, owing to A2 domain mutations, reflects a combination of impaired multimer assembly, increased proteolytic cleavage by ADAMTS-13, and enhanced VWF clearance. In line with the present results, functional studies of some type 2A variants have shown that this VWD type arises from multiple mechanisms, including intracellular retention or degradation of VWF, defective multimerization, impaired regulated storage, and increased proteolysis, with several variants exhibiting overlapping defects in both intracellular and extracellular processes [51].

Notably, the current study expands existing knowledge by linking structural modeling to biochemical and clinical parameters in a well-characterized patient population. To further dissect the mechanistic underpinnings, we employed MD and SMD simulations of 14 VWF A2 variants. These analyses revealed that most mutant A2 domains exhibited attenuated structural flexibility, altered solvent accessibility, and reduced α6 helix protrusion away from the A2 body relative to the WT protein. Notably, under shear stress-mimicking conditions (ie, SMD), only the WT domain displayed significant conformational rearrangement, while all the 14 type 2A mutants remained structurally constrained.

In type 2A, the A2 domain exhibits increased susceptibility to cleavage by ADAMTS-13 due to a conformation that favors interaction with the protease, even in the absence of significant shear stress. A recent study demonstrated that the binding strength between α6-A2 and the spacer-ADAMTS-13 complex, the first step in A2-spacer recognition prior to proteolysis, increases with modest α6 extension, but suddenly decreases beyond 0.25 nm [19]. In our study, the A2 mutants appeared overall unresponsive to shear stress (Figure 5B), unlike their WT counterparts, which seem to acquire greater global floppiness in response to it (Figure 6B) as well as local α6 extendibility. The higher rigidity of A2 mutants in response to perturbation is apparently counterintuitive with respect to a documented increased susceptibility to proteolysis; however, the retained structure and compactness of the α6 helix agree with an optimal response to proteolysis when its elongation is not excessive. According to the simulations reported here, we hypothesize a mechanism that identifies A2-spacer recognition more precisely in mutants than in the WT A2 form, thereby promoting proteolysis more efficiently in the former systems, in agreement with the experimental data. In this context, the α6 helix also demonstrated reduced contact dynamics in all mutants compared with WT, further supporting a pathophysiological model in which loss of dynamic responsiveness contributes to aberrant VWF cleavage processing, in combination with different kinetics of A2 unfolding due to the presence of mutations. Previous studies have shown that there are cases in which the destabilization of the α6 helix in the A2 domain promotes exposure of the ADAMTS-13 cleavage site, even in the folded A2 domain [12], or cases in which the separation of the N- and C-termini promotes a more open conformation that exposes both the ADAMTS-13 binding sites and the scissile bond [52]. Overall, these observations, along with the present work, suggest that different type 2A variants contribute to the phenotype through distinct mechanisms, underscoring the structural and molecular complexity and heterogeneity of this VWD subtype.

The model used in the present work can be seen as a mechanistic view only of the early stages of A2 unfolding and response to shear stress prior to proteolysis; hence, the more complex later stages of total A2 unfolding and its interaction with the ADAMTS-13 metalloprotease domain cannot be considered by this modeling strategy. However, the destabilization of the A2 domain in promoting proteolysis is also locally reflected by a modest increase in the SASA’s proteolytic cleavage site (residues 1603-1606), which is surprisingly already present even in the absence of perturbation (Table 3), as evidenced in the past with other mutants [12]. Despite the apparently low extent of changes in local SASAs, their variation falls between 1 and 6 Å [2], roughly 1 order of magnitude of the minimum amount of SASA variation that identifies buried residues in protein interfaces [53], indicating a clear trend for cleavage site residues to be more exposed in the mutants than in WT A2. In perspective, we also estimated the potential affinity of A2-spacer complexes at the early stages of unfolding by molecular docking under both unperturbed (ie, MD) and perturbed (ie, SMD) conditions, followed by free-energy differences for the complexes computed using MM-PBSA. MD-based docked structures (Figure 7A) led to a rather weak affinity for WT A2 as well as for the p.His1536_Val1537del, p.Arg1597Trp, and p.Gly1629Arg mutants, with the rest of the complexes exhibiting a slightly unfavorable interaction; SMD-based docked structures (Figure 7B) displayed favorable binding only for the WT A2 form (−2.1 kcal/mol), whereas the mutants displayed unfavorable interactions. The overall weak interaction of these complexes, along with the slightly (less than 4 kcal/mol) unfavorable interactions for all the A2 mutants under the effect of perturbations, supports the hypothesis [38] that this interaction alone makes only a modest contribution to proteolysis within the full-length VWF, with functional redundancy between the spacer domain and the C-terminal TSP and CUB domain binding sites of ADAMTS-13. The complete atomic-level picture of these events is beyond the purpose of the present work, which, however, provides indications of a potential future direction, either computational or experimental, for the biophysical characterization of this system.

In conclusion, this study provides a detailed analysis of a large cohort of type 2A VWD associated with A2 domain variants, linking phenotypic, biochemical, and structural findings. Our results reveal that this phenotype results from multiple mechanisms, including reduced VWF:Ag levels, impaired multimerization, increased ADAMTS-13–mediated proteolysis, and enhanced VWF clearance. Structural computational studies show that the point mutations introduced into the protein sequence preserve the overall compactness of A2 at least in the early stages of shear stress, counterintuitively but intriguingly suggesting a crucial role for the rigidification of the α6 helix domain in mastering interactions with the spacer domain of ADAMTS-13. Additionally, intraplatelet VWF assays were valuable for distinguishing between synthesis defects and postsecretion functional abnormalities.

## Supplementary material

Supplementary Figure **Multimeric pattern of type 2A VWD with A2 domain mutations.** Examples are shown for type 2A VWD patients harboring A2 domain mutations. The multimeric pattern is displayed by densitometric analysis alongside the corresponding electrophoresis gel (lane 1 of both gels: pooled normal sample; lane 3 of left gel and lanes 4–5 of right gel: type 2A VWD patients).
