# Supplementary material for: Deep molecular modeling and mechanistic insights into type 2A von Willebrand disease with von Willebrand factor A2 domain mutations
Source: Res Pract Thromb Haemost. 2025 Oct 22;9(7):103233. doi: 10.1016/j.rpth.2025.103233 (PMC12657285; doi:10.1016/j.rpth.2025.103233)
Supplement: Supplementary Figure — Multimeric pattern of type 2A VWD with A2 domain mutations. Examples are shown for type 2A VWD patients harboring A2 domain mutations. The multimeric pattern is displayed by densitometric analysis alongside the corresponding electrophoresis gel (lane 1 of both gels: pooled normal sample; lane 3 of left gel and lanes 4–5 of right gel: type 2A VWD patients). [file mmc1.pdf]

Supplementary Figure

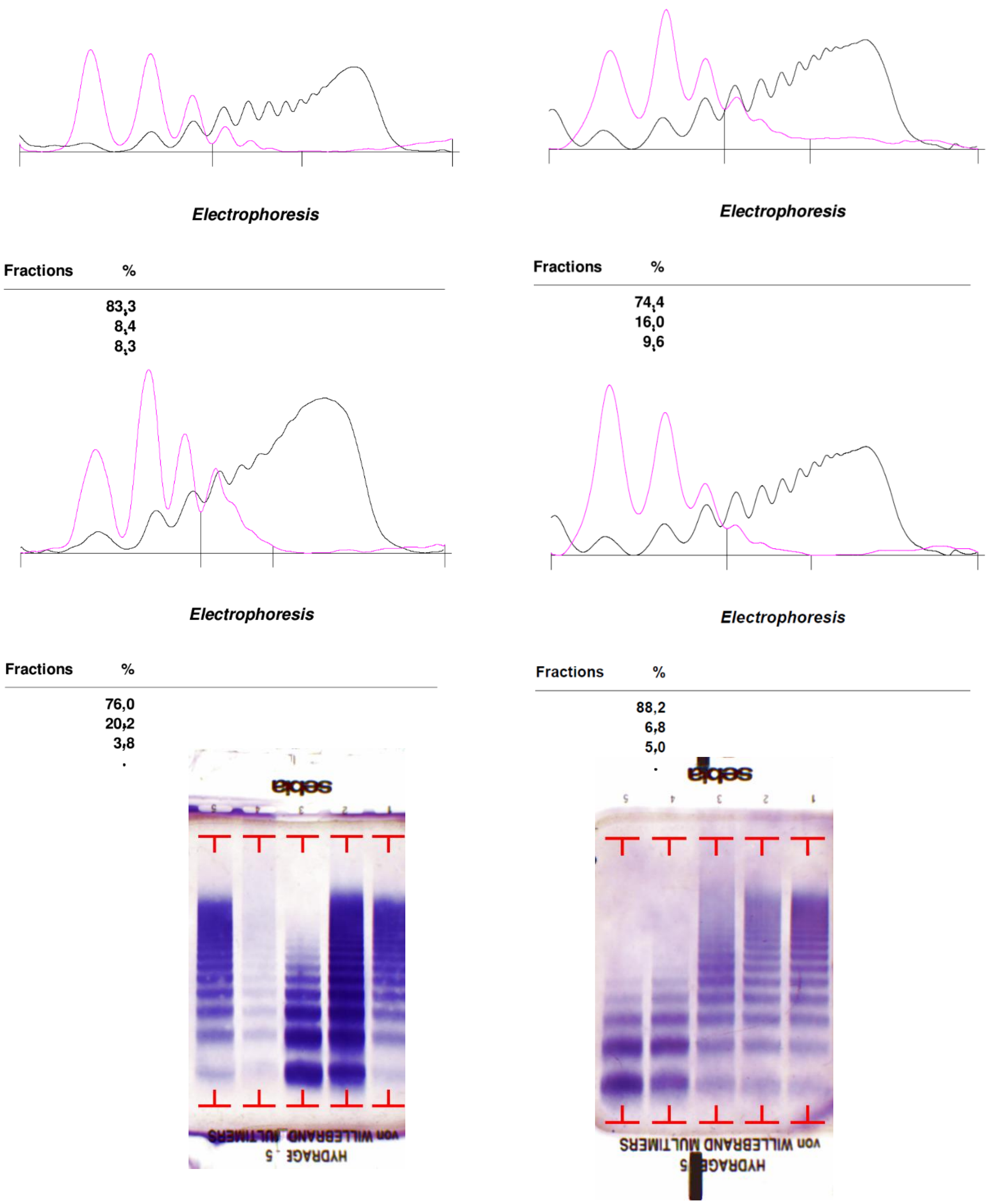

Supplementary Figure. Multimeric pattern of type 2A VWD with A2 domain mutations. Examples are shown for type 2A VWD patients harboring A2 domain mutations. The multimeric pattern is displayed by densitometric analysis alongside the corresponding electrophoresis gel (lane 1 of both gels: pooled normal sample; lane 3 of left gel and lanes 4–5 of right gel: type 2A VWD patients).
